# Biofortification Technology for the Remediation of Cadmium-Contaminated Farmland by the Hyperaccumulator *Sedum alfredii* under Crop Rotation and Relay Cropping Mode

**DOI:** 10.3390/toxics10110691

**Published:** 2022-11-15

**Authors:** Haiyun Xie, Jiuzhou Chen, Yabei Qiao, Kuan Xu, Zhi Lin, Shengke Tian

**Affiliations:** MOE Key Laboratory of Environmental Remediation and Ecological Health, College of Environmental and Resource Sciences, Zhejiang University, Hangzhou 310058, China

**Keywords:** cadmium, *Sedum alfredii*, phytoextraction, biofortification

## Abstract

Soil cadmium (Cd) extraction for hyperaccumulators is one of the most important technologies for the remediation of Cd-contaminated farmland soil. However, a phytoremediation model using a single hyperaccumulator cannot guarantee normal agricultural production in contaminated areas. To solve this problem, a combination of efficient remediation and safe production has been developed. Based on two-period field experiments, this study explored the effect of biofortification on soil Cd remediation using the fruit tree *Sedum alfredii* Hance and oil sunflower crop rotation and relay cropping mode. BioA and BioB treatments could markedly improve the efficiency of Cd extraction and remediation, and the maximum increase in Cd accumulation was 243.29%. When BioB treatment was combined with papaya–*S. alfredii* and oil sunflower crop rotation and relay cropping mode, the highest soil Cd removal rate in the two periods was 40.84%, whereas the Cd concentration of papaya fruit was lower than safety production standards (0.05 mg/kg). These results demonstrate that biofortification measures can significantly improve the Cd extraction effect of *S. alfredii* crop rotation and relay cropping restoration modes, which has guiding significance for Cd pollution remediation and safe production in farmland.

## 1. Introduction

Soil cadmium (Cd) pollution has become a major problem worldwide owing to its high mobility in the environment and toxicity to humans [1]. National Soil Pollution Survey Bulletin shows that the over-standard rate of soil pollution points in China is 19.4% [2]. In the process of agricultural production, with the application of pesticides and organic fertilizers, heavy metals will soon enter the farmland. Through food chain enrichment, these soil heavy metals may eventually enter the human body, endangering human health. Therefore, the repair of Cd-contaminated soils and the restoration of safe cultivation are urgently needed.

Existing enhanced remediation technologies can be broadly classified into physical, chemical, and biological methods, among which phytoremediation is an environmentally friendly, economically feasible, sustainable, and disposable option [3,4]. However, the use of hyperaccumulators for phytoremediation has problems, such as long repair cycles and small biomass. Under the current situation of “large population and small land” in China, using phytoremediation on a large scale is unrealistic. Therefore, using hyperaccumulators to repair farmlands while ensuring normal agricultural production activities is key to applying remediation in agricultural practice. *S. alfredii* is a native zinc (Zn)/Cd hyperaccumulator and lead (Pb)-enriched plant in China, which was first discovered in an ancient Pb-Zn mining area in southeastern of China [5]. Through natural evolution, the Cd concentration in the stems and leaves of *S. alfredii* can be as high as 9000 mg/kg [6]. With strong environmental adaptability, it is an ideal phytoremediation material with perennial, asexual reproduction, larger biomass and suitable for mowing. Meanwhile, the harvested plants can be disposed of harmlessly. In the actual restoration process, planting *S. alfredii* alone has high labor costs and no economic benefits. In order to ensure the income of farmers, combined with bioaugmentation remediation measures, the *S. alfredii* crop rotation/intercropping/relay cropping mode is usually adopted, which can not only reduce the remediation cost, but also improve the remediation efficiency of *S. alfredii*. In addition, this method significantly shortens the remediation period, and achieves the goal of phytoremediation coupled with agro-production.

In soil pollution remediation, biofortification measures include microbial and plant biostimulant enhancement. In fact, excess Cd in the soil reduces plant uptake of nutrients (P, Fe, Zn, and Mn), whereas microorganisms can promote the increase of these nutrients in plants and prevent the adverse effects of Cd [7]. For example, the synergistic effect of *Bacillus subtilis* and other microorganisms can effectively increase plant yields [8]. As an endogenous plant hormone, brassinolide is widely used to reduce the adverse effects of Cd in crops. Several studies have shown that the application of brassinolide can improve the toxic effects of Cd [9,10,11,12]. Biostimulants include protein hydrolysates and amino acids, seaweed extracts, humic acids, chitin, chitosan and its derivatives, and microbial agents [13]. Alginate fish protein fertilizer is an amino acid water-soluble fertilizer that is rich in polypeptides, amino acids, and alginate. Its exogenous application can play a role in the rooting and strengthening of seedlings. However, there are few studies on the effects of these biofortification measures on the growth, Cd uptake, and accumulation of *S. alfredii*.

Therefore, in this study, using fruit tree–*S. alfredii*–oil sunflower crop rotation and relay cropping planting mode, the effects of different biofortification measures on the Cd remediation efficiency were clarified via two-period field experiments. By analyzing the changes of plant biomass, plant Cd concentration, soil Cd concentration, soil Cd removal rate and fruit Cd concentration, the mechanism of biofortification measures combined with crop rotation and relay cropping mode affecting plant absorption and accumulation of Cd was preliminarily elucidated, aiming to provide reference for the remediation of Cd-contaminated farmland and the safe cultivation of crops.

## 2. Materials and Methods

### 2.1. Experimental Soil and Materials

Field experiments were conducted at the Foshan Agricultural Technology Extension Center (112.86° E, 43.54° N). The classification standard of soil Cd pollution grade is risk screening value. Soil samples were taken from the surface layer (0–20 cm). The physical and chemical properties of the experimental soil are shown in Table 1. The exogenous additives, components, and application methods used in this study are listed in Table 2.

### 2.2. Design of Field Experiments

The experiment comprised three rotation and intercropping systems: grapefruit–*S. alfredii*–oil sunflower, papaya–*S. alfredii*–oil sunflower, and fig–*S. alfredii*–oil sunflower. *S. alfredii* and oil sunflower plants were cultivated in rotation with grapefruit/papaya/fig as the intercrops. The fruit tree seedlings were 3-year-old seedlings, grown one year in advance, in the middle of each plot of single-row planting. The experiment was divided into two periods. In the first cultivation period, oil sunflower was intercropped from June to September 2018, and *S. alfredii* was intercropped from October 2018 to January 2019. In the second period of the experiment, oil sunflower was intercropped from June to September 2019, and *S. alfredii* was intercropped from October 2019 to January 2020.

Two composite treatments were applied to *S. alfredii* (Table 3), each set up in three plots with an area of 2.5 m × 12 m. Soil and plant samples were collected at the end of the two periods.

### 2.3. Sample Collection

For the collection of non-rhizosphere soil in each plot: an S-shaped sampling method was applied to dig a 0–20 cm layer of soil for the determination of physicochemical properties.

For the collection of rhizosphere soil in each plot: parts of the root system were pulled out of plants showing the same growth in the fields. After shaking off the rhizosphere soil clumps with low adhesion, the soil near the root system was collected using tweezers as was the rhizosphere soil (approximately 100 g) for later analysis of the physicochemical properties.

For the plant collection: Plants showing the same growth rate in the plots were selected, and whole plants of *S. alfredii*, maize, and oil sunflower were dug out. For fruit trees, 3–4 branches, 3–4 fruits, and a few roots were collected from each tree. After returning to the laboratory, the plants were immersed in a 20 mmol/L EDTA-2Na solution for 10 min and washed several times with deionized and ultrapure water. The plants were divided into parts. *S. alfredii* was divided into shoot and root; oil sunflower into the grain, receptacle, stem, leaf, and root; and maize into grain, cob, stem, leaf, root, and fruit. After drying on absorbent paper, the collected parts were placed in an oven at 110 °C for 30 min and then baked at 65 °C until a constant weight was achieved, followed by the determination of their physicochemical properties.

### 2.4. Determination of Soil Physicochemical Properties

The soil samples were laid flat and dried in a cool, ventilated place. Impurities such as stones were removed through a 2 mm sieve, followed by a 1 mm and 0.149 mm sieve. The 0.50 g soil sample passed through a 0.149 mm sieve was digested using concentrated H_2_SO_4_–HClO_4_, and total phosphorus in the soil was determined using a continuous flow analyzer (Skalar SAN++, The Netherlands). A total of 5.00 g of the 2 mm sieved soil sample was weighed and added to CO_2_-free water (water:soil ratio 2.5:1) and stirred vigorously. After standing for 30 min, the pH was determined using a portable pH meter (STARTER 300, USA). A 2.50 g A 1 mm air-dried sample was weighed and extracted with a 0.5 mol/L NaHCO_3_ solution, and the available soil phosphorus content was determined using a continuous flow analyzer. A total of 2.00 g of the 1 mm sieved soil sample was weighed, and available nitrogen content was determined using the alkaline hydrolysis diffusion method. A total of 5.00 g of the 2 mm sieved soil sample was weighed and added to 15 mL DTPA-TEA, soaked, and shaken for 2 h. The leaching solution was then filtered using qualitative filter paper and diluted, and the soil available Cd concentration in the solution was determined using inductively coupled plasma mass spectrometry (ICP-MS, Thermo, USA). A 0.149 mm sieved soil sample (0.100 g) was weighed and soaked in concentrated HNO_3_–HClO_4_–HF (solution volume ratio 5:1:1) for 8 h. The soil sample solution was then placed in a high-temperature oven and boiled at 180 °C for 10 h. After acid capture, filtration, and dilution, the sample was passed through inductively coupled plasma mass spectrometry (Thermo Scientific™ iCAP™ RQ ICP-MS) to determine the total Cd concentration in the soil digestion solution. Quality control of the composition analysis was performed using a soil standard substance GBW07405 (GSS-5). A total of 5.00 g of the 1 mm sieved soil sample was weighed, added to 50 mL of neutral NH_4_OA solution, and shaken for 30 min. After filtration with qualitative filter paper, soil available potassium was determined using a flame photometer (M410, SHERWOOD, England).

### 2.5. Determination Plant Cd Concentration

The dried plants were weighed, and the dry weight biomass of different parts was recorded. After being cut into pieces with stainless-steel scissors, they were placed in a ball mill (RETSCH MM400, Germany, zirconia grinding tank) for crushing. A 0.05 g plant powder sample was added to a glass digestion tube, and concentrated HNO_3_–H_2_O_2_ was added. The solution was incubated at 120 °C until colorless or light yellow and clear. After filtration and dilution, the Cd concentration in the digestion solution was determined using ICP-MS. Quality control of composition analysis using spinach standard substance GBW10015 (GSB-6).

## 3. Results and Analysis

### 3.1. Growth and Cd Uptake and Accumulation in S. alfredii Shoots

**Figure 1:**
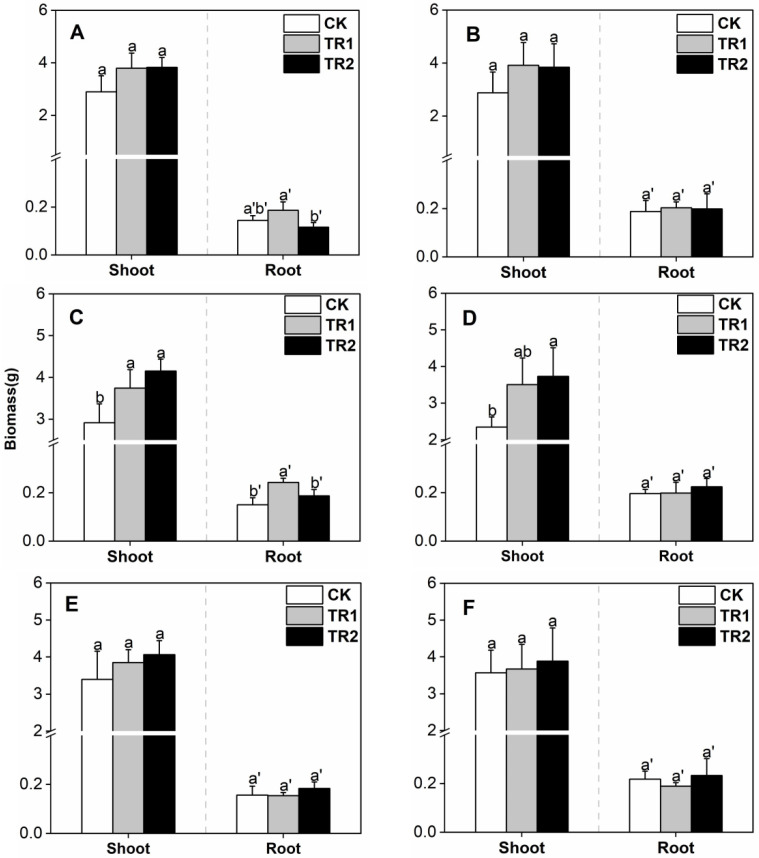
*S. alfredii* biomass. (**A**) First period intercropping papaya, (**B**) Second period intercropping papaya, (**C**) First period intercropping grapefruit, (**D**) Second period intercropping grapefruit, (**E**) First period intercropping fig, (**F**) Second period intercropping fig. The different letters above the column indicate the significant differences among different treatments at *p* < 0.05.

In the CK treatment, the shoot biomass of *S. alfredii* was in the range of 2.35–3.57 g (Figure 1). The addition of the two combined treatments significantly increased the shoot biomass of *S. alfredii* only in intercropping with grapefruit. Compared with CK, among them, the effect of the BioB treatment on shoot biomass was better and increased the shoot biomass by 42.41 and 58.92% in the first and second periods, respectively.

**Figure 2:**
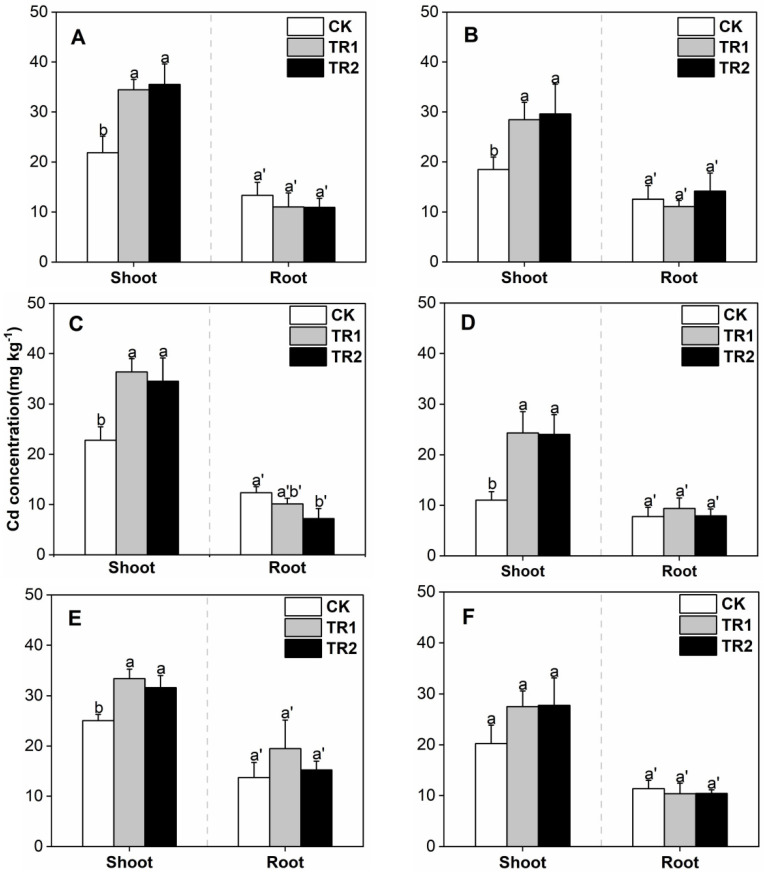
*S. alfredii* Cd concentration. (**A**) First period intercropping papaya, (**B**) Second period intercropping papaya, (**C**) First period intercropping grapefruit, (**D**) Second period intercropping grapefruit, (**E**) First period intercropping fig, (**F**) Second period intercropping fig. The different letters above the column indicate the significant differences among different treatments at *p* < 0.05.

Under different intercropping patterns, the Cd concentrations in *S. alfredii* shoots treated with CK were between 11.02–25.05 mg/kg (Figure 2). Both combined treatments significantly increased the Cd concentration in the shoots of *S. alfredii*, and the effects were similar. Compared with CK, the minimum increase in BioA treatment was 33.16% and the maximum was 120.72%. The minimum increment in BioB treatment was 26.21%, and the maximum was 118.15%. In addition, the Cd concentration in *S. alfredii* shoots was significantly lower than that during the first period.

**Figure 3:**
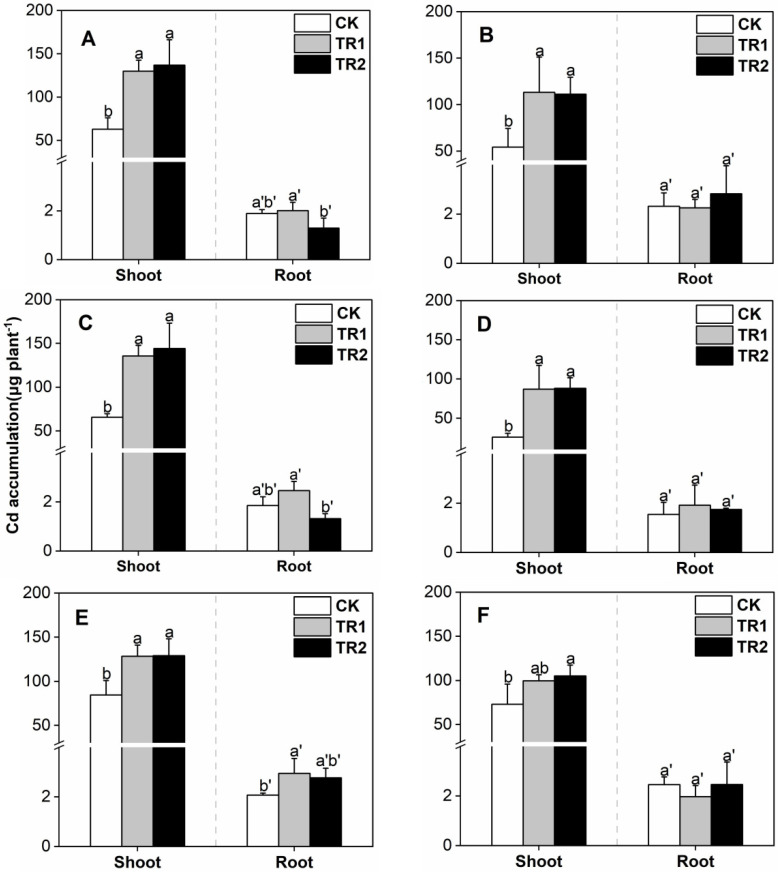
*S. alfredii* Cd accumulation. (**A**) First period intercropping papaya, (**B**) Second period intercropping papaya, (**C**) First period intercropping grapefruit, (**D**) Second period intercropping grapefruit, (**E**) First period intercropping fig, (**F**) Second period intercropping fig. The different letters above the column indicate the significant differences among different treatments at *p* < 0.05.

Under different intercropping patterns, in the CK treatment, Cd accumulation in the *S. alfredii* shoots was 25.88–84.54 mg/kg (Figure 3). Both the combined treatments significantly increased Cd accumulation in the shoots of *S. alfredii*, and the effects were similar. Compared with CK, the increase in microbial agent + CFB treatment was 87.15–135.54% and was 88.24–144.18% in the BioB treatment. With increasing planting years, there was no significant change in the shoot biomass of *S. alfredii*. The decrease in Cd concentration led to a decrease in Cd accumulation.

### 3.2. Growth and Cd Uptake and Accumulation in Oil Sunflower

**Figure 4:**
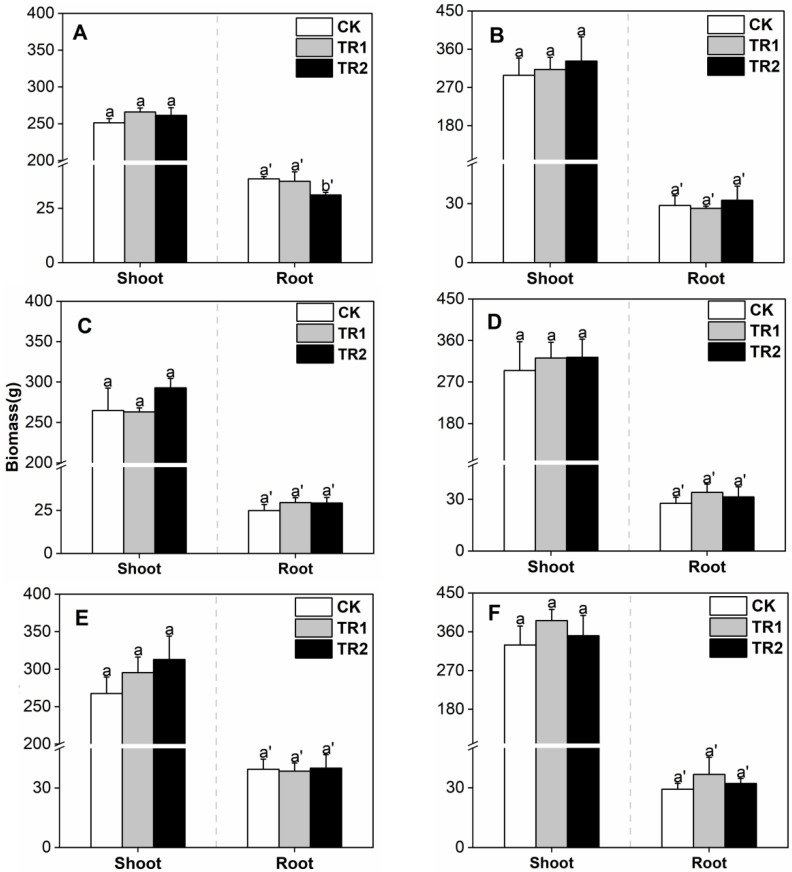
Oil sunflower biomass. (**A**) First period intercropping papaya, (**B**) Second period intercropping papaya, (**C**) First period intercropping grapefruit, (**D**) Second period intercropping grapefruit, (**E**) First period intercropping fig, (**F**) Second period intercropping fig. The different letters above the column indicate the significant differences among different treatments at *p* < 0.05.

Compared with CK, the two treatments had no significant effect on the shoot biomass of oil sunflower (Figure 4).

**Figure 5:**
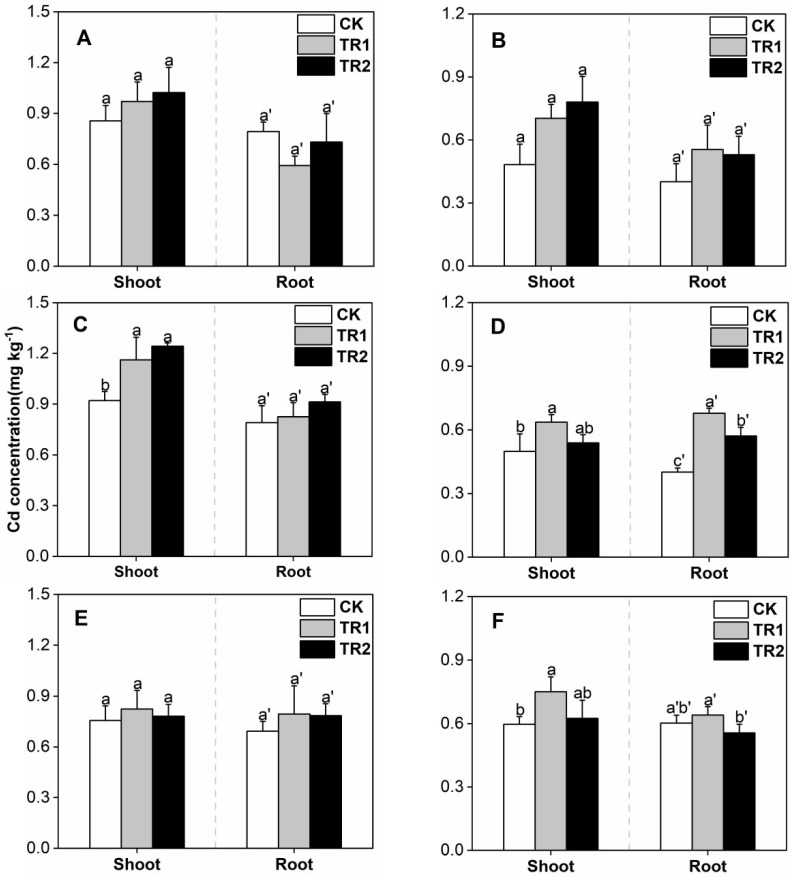
Oil sunflower Cd concentration. (**A**) First period intercropping papaya, (**B**) Second period intercropping papaya, (**C**) First period intercropping grapefruit, (**D**) Second period intercropping grapefruit, (**E**) First period intercropping fig, (**F**) Second period intercropping fig. The different letters above the column indicate the significant differences among different treatments at *p* < 0.05.

Under CK treatment, the Cd concentration in the shoot of oil sunflower was 0.48–0.92 mg/kg (Figure 5). Compared with CK, the addition of the two combined treatments could increase the Cd concentration in the shoots of oil sunflower to a certain extent, with an increase of 3.35 to 61.82%. The Cd concentration of the sunflower oil in the second period was lower than that in the first period.

**Figure 6:**
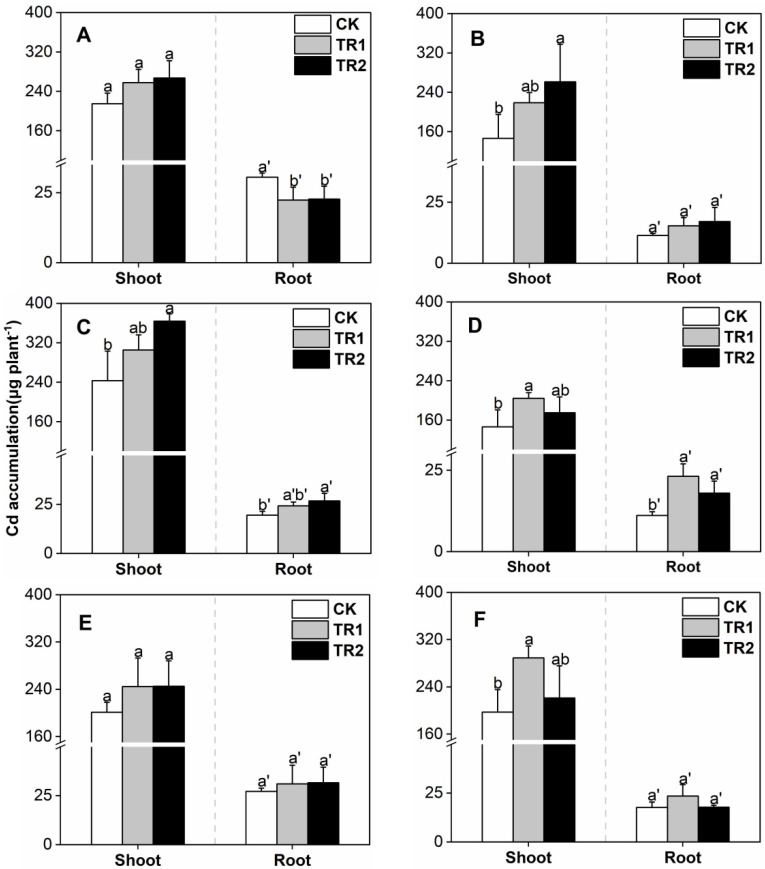
Oil sunflower Cd accumulation. (**A**) First period intercropping papaya, (**B**) Second period intercropping papaya, (**C**) First period intercropping grapefruit, (**D**) Second period intercropping grapefruit, (**E**) First period intercropping fig, (**F**) Second period intercropping fig. The different letters above the column indicate the significant differences among different treatments at *p* < 0.05.

Under the CK treatment, Cd accumulation in oil sunflower shoots was 146.22–243.29 mg/kg (Figure 6). BioA treatment significantly increased Cd accumulation in the shoots of oil sunflowers. Compared with CK, the increase of BioA treatment was 19.92–49.59%, and the increase of BioB treatment was 12.09–78.58%. Compared with *S. alfredii*, oil sunflower has a larger unit planting area and relatively insufficient Cd extraction capacity, but in the oil sunflower-*S. alfredii* intercropping system, oil sunflowers can provide shading conditions for *S. alfredii* while extracting Cd, which is an excellent combination of soil Cd extraction materials.

### 3.3. Soil Cd Concentration and Removal Rate

**Figure 7:**
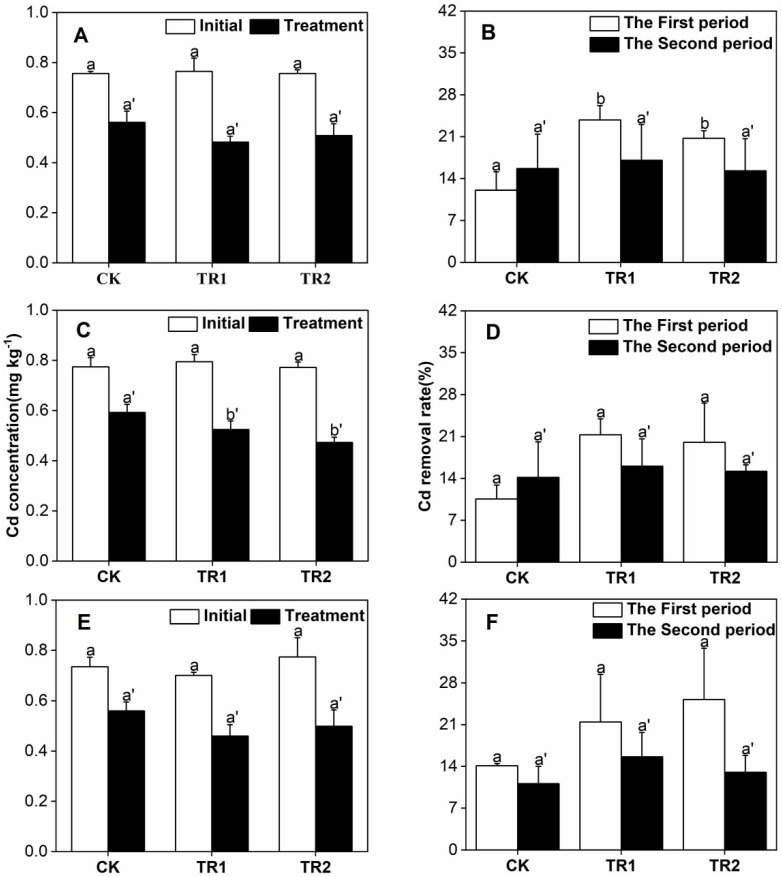
Soil Cd Concentration and Removal Rate. (**A**) First period intercropping papaya, (**B**) Second period intercropping papaya, (**C**) First period intercropping grapefruit, (**D**) Second period intercropping grapefruit, (**E**) First period intercropping fig, (**F**) Second period intercropping fig. The different letters above the column indicate the significant differences among different treatments at *p* < 0.05.

Before and after planting, the classifications standard of soil Cd pollution grade all risk screening values. After two periods of planting, when relay cropped with grapefruit, the soil Cd concentration in BioA and BioB treatments was significantly different from that in the CK treatment. Compared with the initial soil Cd concentration, it decreased by 23.48%, 33.97% and 28.72%, respectively. After two planting periods, the soil Cd removal rate in the CK treatment was 23.48–25.82% (Figure 7) and intercropping with grapefruit and treatment with BioB showed the highest Cd removal rate of 38.72%. 

### 3.4. Concentration of Cd in Fruits

**Figure 8:**
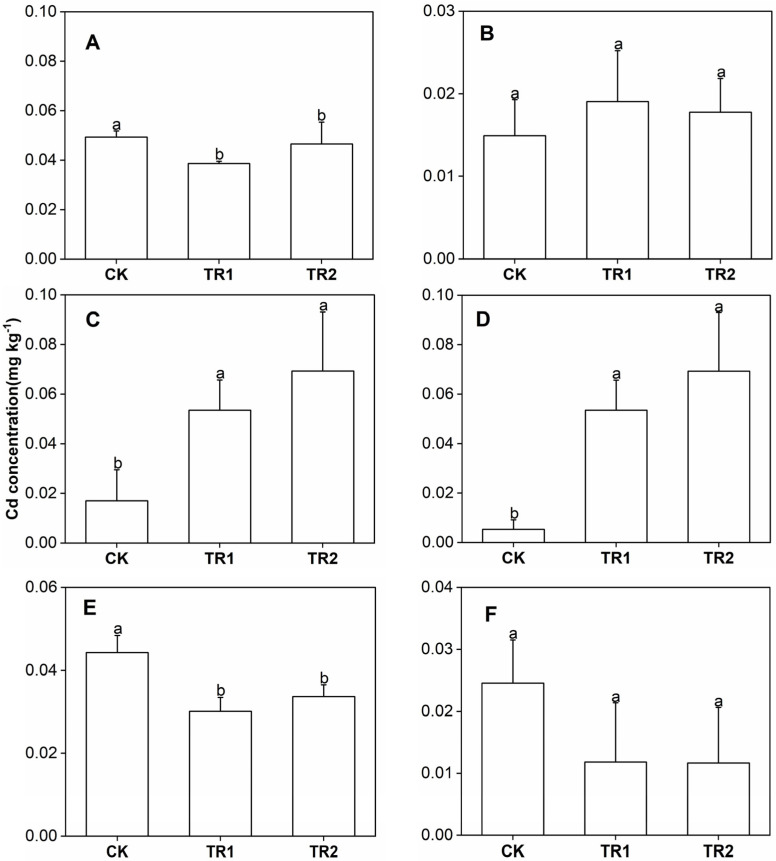
Fruit Cd concentration. (**A**) First period intercropping papaya, (**B**) Second period intercropping papaya, (**C**) First period intercropping grapefruit, (**D**) Second period intercropping grapefruit, (**E**) First period intercropping fig, (**F**) Second period intercropping fig. The different letters above the column indicate the significant differences among different treatments at *p* < 0.05.

Under the CK treatment, the Cd concentration in fruits was 0.01–0.05 mg/kg (Figure 8). The two treatments did not significantly increase the Cd concentration in papaya and fig fruits but significantly increased the Cd concentrations in grapefruit. The highest Cd concentration was 0.07 mg/kg, which exceeded the limit of Cd (0.05 mg/kg) in the national food safety standard. Therefore, it is not recommended to plant grapefruit in farmlands with soil Cd risk screening values and above.

## 4. Discussion

### 4.1. Effects of Bioaugmentation on Plant Growth

An important indicator of plant growth, biomass largely measures the tolerance potential of plants to heavy metal stress [14,15]. Bioaugmentation exerted different effects on the biomass of *S. alfredii* and oil sunflower. BioA and BioB applications synergistically promoted the plant–soil–microorganism system. In Cd-contaminated farmland, under the fruit trees and *S. alfredii* and oil sunflower crop rotation and relay cropping mode, BioA and BioB significantly increased the shoot biomass of *S. alfredii*, which was consistent with the findings of previous studies [16,17,18]. *B. subtilis* can reduce Cd toxicity by increasing water absorption and reducing electrolyte leakage, thereby promoting plant growth [19]. Under Cd stress, 24-EBL significantly increased plant growth and biochemical parameters, including stem length, stem dry weight, and total chlorophyll content [20]. Sodium alginate significantly promotes the growth of *S. alfredii*, greatly promoting the absorption of Cd by *S. alfredii* shoots [16]. Furthermore, fish protein fertilizer is superior to commonly used manure and NPK fertilizers in promoting the healthy growth of plants [21]. In the second period of grapefruit–*S. alfredii*–oil sunflower crop rotation and relay cropping, the biomass of *S. alfredii* treated with BioB increased the most, reaching 58.92%. However, bioaugmentation exerted no significant effect on the shoot biomass of oil sunflower, which differs from the previous findings [17]. This may be because of the fact that the growth of oil sunflower is affected by experiment duration and the soil climate.

### 4.2. Effects of Bioaugmentation on Cd Uptake and Accumulation in Plants

The main route for Cd to enter plants is through the roots absorbing Cd from soil or water, or the leaves absorbing Cd from the atmosphere [22,23]. Under Cd stress, plants exhibit a series of physiological responses to detoxification and heavy metal enrichment. Previous studies have shown that the main response mechanisms of plants under Cd stress are root absorption, compartmentalization, chelation, antioxidation, stress, and osmotic regulation [23,24]. Among these, root function, compartmentalization, and chelation of plants are the dominant factors in the process of Cd enrichment.

Compared with the BioA treatment, the BioB treatment had the best effect on Cd uptake and accumulation in *S. alfredii* and on Cd accumulation in oil sunflowers. Brassinolide can have many effects on plants, including increasing the activity of RuBP carboxylase in plants, accelerating the CO_2_ fixation rate in leaves, increasing chlorophyll and protein content, superoxide dismutase (SOD) and peroxidase (POD) activity in leaves, and reducing malondialdehyde content and electrolyte leakage rate in leaves [25]. Thus, brassinolide promotes photosynthesis and improves the water use efficiency of plants, and ultimately enhances the tolerance of plants to Cd. Sodium alginate contains a large number of free carboxyl groups that can react with metal ions. During adsorption, heavy metal ions exchange with Na^+^ ions in sodium alginate [26]. Therefore, BioB can comprehensively improve the Cd extraction efficiency of plants by increasing plant enzyme activity, improving disease resistance, enhancing the absorption of heavy metal ions, and increasing nutrient elements.

### 4.3. Effects of Different Remediation Modes on Soil Cd Removal Rate

Plants grown in contaminated soil must be tolerant to a variety of heavy metals to ensure good phytoremediation efficiency [27]. In this study, bioaugmentation technology was used to improve the biomass or Cd absorption capacity of remediation plants, thereby improving the removal rate of Cd in soil. After two periods of planting, in the papaya–*S. alfredii*–oil sunflower crop rotation and relay cropping mode, the BioB treatment had the highest soil Cd removal rate, at 40.84%. Papaya has a larger shading area on the ground and a deeper root system; thus, it does not strongly compete with *S. alfredii* in its niche, and therefore may be more conducive to the growth of *S. alfredii*.

With restoration, soil Cd concentration gradually decreased, and the absorption and accumulation rates of *S. alfredii* also decreased. Therefore, Cd accumulation in the shoots of *S. alfredii* during the second period was significantly lower than that during the first period. In the remediation plants, although Cd accumulation in the shoots of oil sunflower was significantly higher than that of *S. alfredii*, the unit area of oil sunflower was also much larger than that of *S. alfredii*. Therefore, the restorative effect of *S. alfredii* oil was higher than that of oil sunflower. It can take a long time to remediate contaminated soil by only planting hyperaccumulators thus occupying farmland soil production resources. Crop rotation and relay cropping using hyperaccumulating plants that absorb fewer heavy metals or transport less to edible parts, can produce agricultural products that meet safety standards while also repairing contaminated soil. Therefore, this could be a more economical and reasonable treatment and utilization method without intermittent agricultural production.

### 4.4. Effect of Biofortification on Fruit Production Safety

Cd concentration in crops is affected by biofortification. The addition of *B. subtilis* increases the Cd concentration in soil but can reduce the Cd concentration in rape [28]. The application of brassinolide reduces the lipid peroxidation of rice, promotes the uptake of nitrogen and phosphorus, and increases the photosynthetic rate, thereby improving rice growth and yield and effectively reducing the concentration of Cd in rice grains [29]. By absorbing and transporting amino acids and small peptides hydrolyzed by proteins, plant roots regulate plant metabolism and physiological and biochemical reactions, promote seed germination and root development, enhance nutrient absorption, improve plant stress resistance, and increase crop yield [30,31]. In addition, regardless of whether the fruit was contaminated, the fruit Cd concentration was affected by the soil Cd concentration and fruit variety [32,33]. The common fruits used herein, papaya, grapefruit, and fig, can be safely produced while repairing farmland soil, ensuring the benefits of agricultural production. Following two consecutive periods of remediation, the soil reached the safety production standard. However, although the Cd concentrations of papaya and fig fruit were lower than national food safety standards; the Cd concentration in grapefruit was greater than 0.05 mg/kg. Therefore, only relay-cropping papaya and figs were in line with safe production.

## 5. Conclusions

The two-period field experiments of fruit tree–*S. alfredii*–oil sunflower crop rotation and relay cropping were carried out by biofortification measures. The results showed that the two combined treatments significantly increased the shoot biomass of *S. alfredii* only when relay cropped with grapefruit. Among them, BioB treatment had the best effect in the second period, which increased by 58.92% compared with CK. Under relay cropping with different fruit trees, the two combined treatments significantly increased the Cd concentration and accumulation in the shoot of *S. alfredii*. Among them, BioA treatment showed the best effect on Cd concentration, with the maximum increase of 120.72% compared with CK. BioB treatment had the best effect on Cd accumulation, with the maximum increase of 144.18% compared with CK. The two combined treatments could greatly improve the efficiency of Cd extraction and remediation of *S. alfredii*, and the increase in Cd accumulation was as high as 243.29%. When the intercropping fruit tree was papaya and BioB was applied, the soil Cd removal rate reached the highest (40.84%) in the two periods; the Cd concentration in the fruits did not exceed the national standard. Therefore, BioB treatment combined with papaya–*S. alfredii*–oil sunflower crop rotation and relay cropping can be promoted as an efficient and economical extraction Cd system.

## Figures and Tables

**Table 1:** Soil physical and chemical properties of experimental field.

Physicochemical Properties	Available Nitrogen (mg/kg)	Total Phosphorus (g/kg)	Available Phosphorus (mg/kg)	Available Potassium (mg/kg)	Total Cd (mg/kg)	pH
Soil	110.62	1.35	151.67	138.12	0.67	6.59

**Table 2:** Exogenous substance information.

Exogenous Substance	Components	Application Method
Compound special fertilizer B (BioA)	Polypeptide 170 g/L, free amino acid 50 g/L, organic nitrogen 32 g/L, alginate120 g/L, Acid Soluble Protein 150 g/L, Zn + Mn + B + Fe 5 g/L, *Bacillus subtilis*	Diluted 200–500 times and applied by watering or spraying
Exogenous hormone	24-epibrassinolide (24-EBL)	24-EBL diluted 2000–3000 times and applied by spray

**Table 3:** Field treatment program.

Group	Treatment
CK	Additive free
TR1	BioA
TR2	BioB (24-EBL + BioA)

## Data Availability

The data presented in this study are available upon request from the corresponding author.
